# Linear and Nonlinear Elastic Properties of Polystyrene-Based Nanocomposites with Allotropic Carbon Fillers and Binary Mixtures

**DOI:** 10.3390/polym14245462

**Published:** 2022-12-13

**Authors:** Andrey V. Belashov, Anna A. Zhikhoreva, Olga A. Moskalyuk, Yaroslav M. Beltukov, Irina V. Semenova

**Affiliations:** 1 Ioffe Institute, Russian Academy of Sciences, 26, Polytekhnicheskaya, St. Petersburg 194021, Russia; 2 Laboratory of Polymer and Composite Materials «SmartTextiles», IRC–X-ray Coherent Optics, Immanuel Kant Baltic Federal University, 14, A.Nevsky str., Kaliningrad 236041, Russia

**Keywords:** polymer nanocomposites, carbon nanofillers, binary fillers, elastic properties, dynamic mechanical analysis, nonlinear elastic moduli

## Abstract

We report measurements of linear and nonlinear elastic properties of polystyrene-based nanocomposites with six types of nanofillers, including single and binary mixtures of allotropic carbon nanoparticles. Composite samples were fabricated by the same technology and contained the same filler concentration (5% wt.), which allowed for a direct comparison of their properties. It was shown that the most significant variations of linear and nonlinear elastic properties occur in different nanocomposites. In particular, the most pronounced enhancements of linear elastic moduli (in about 50%) obtained in tensile and flexural tests and in dynamic mechanical analysis were recorded in the sample filled with spherical fullerene nanoparticles. While the most profound rise of absolute values of nonlinear elastic moduli (tens of times) was obtained in the sample filled with the mixture of carbon nanotubes and graphene. The observed tendencies demonstrated the synergistic effect of fillers of different dimensionality on the elastic properties of nanocomposites.

## 1. Introduction

For years polymers were being reinforced with various fillers that allowed for attaining unique mechanical and physical properties in the resulting composite materials, see, e.g., [1,2] and references therein. One of the broad classes of fillers used are nanofillers, which can be classified by dimensionality: zero-dimensional (0D) spherical particles, e.g., SiO${}_{2}$ particles or fullerenes, one-dimensional (1D) tubular fillers, for instance, carbon or halloysite nanotubes, and two-dimensional (2D) platelets, such as layered silicate or graphene. Nanocomposites demonstrate enhanced mechanical properties as compared to host polymers and provide an opportunity to tune these properties by choosing a proper filler and by varying its concentration in the polymer matrix [3]. The improved mechanical properties of nanocomposites are partly due to an extended interfacial area between the polymer matrix and filler particles. It was observed that nanoparticles with a high surface-to-volume ratio provide the most significant reinforcement [4].

A class of nanofillers widely utilized in polymer composites is allotropic forms of carbon, including carbon black, fullerenes, graphene, carbon nanotubes and nanofibers. These fillers were shown to provide prominent improvements in the mechanical performance of the materials and their thermal and electrical properties [5,6,7,8,9]. The key points in achieving the desired properties of the composite are good dispersion and homogeneous distribution of filler particles in the polymer matrix. Substantial aggregation can cause aggravated performance of the material. Currently, attempts are being made to use hybrid fillers containing a mixture of two or more nanofillers with different dimensionality [10,11,12]. Hybridization was suggested to be beneficial for the disintegration of agglomerates, was shown to facilitate the formation of an interconnected structure of filler particles in the matrix [5] and to provide a synergistic effect on the enhancement of mechanical performance, see [13,14] and references therein. The utilization of hybrid fillers can provide a combination of the advantages of each component along with the diminishment of their drawbacks.

Traditionally, the analysis of the mechanical performance of materials is based on a set of tests providing data on their linear elastic properties. The certified test methods include tensile, flexural and compression tests and dynamic mechanical analysis. Currently, the nonlinear elastic behavior of materials becomes increasingly important in view of their performance under various dynamic loads. However, no test methods have been certified for the analysis of the nonlinear elastic properties of materials. Most common approaches utilize sets of third-order elastic moduli characterizing the nonlinearity of a stress-strain relationship. The most frequently used set is based on Murnaghan’s approximation [15] and contains three nonlinear, third-order moduli (l,m,n) and two linear, second-order, Lamé moduli (λ, μ). This approach was widely applied for the characterization of the nonlinear elasticity of various materials: metals and alloys [16,17,18], rocks [19], crystals [20], etc. Investigations of nonlinear elastic properties of composite materials are quite rare so far [21,22], are performed on randomly chosen materials and do not provide systematic data on the dependence of nonlinear properties of composites on the filler type and concentration.

In this research, we performed a complex comparative analysis of linear and nonlinear elastic properties of polystyrene-based nanocomposites with three carbon nanofillers of different dimensionality: 0D fullerenes, 1D carbon nanotubes and 2D graphene platelets, and their binary mixtures. Composite samples were fabricated by the same technology and contained the same filler concentration (5% wt.) to allow direct comparison of their properties.

## 2. Materials and Methods

### 2.1. Fabrication of Nanocomposite Samples

The grained 585 polystyrene (Nizhnekamskneftekhim, Nizhnekamsk, Russia) was used as a base polymer matrix. For nanofillers, we used allotropic forms of carbon: fullerenes C${}_{60}$ (MST-Nano, St.Petersburg, Russia), multiwalled carbon nanotubes (CNT) Taunit-M (NanoTechCenter, Tambov, Russia), graphene (Gr) (Rusgraphene, Protvino, Russia) and their binary mixtures. According to the data provided by manufacturers, filler particles had the following parameters. CNTs: diameter 10–30 nm, length ≥2μm and specific surface area ≥ 270 m${}^{2}$/g. Graphene platelets: monolayer thickness 1–4 nm, size 10–100 μm and monolayer content ≥80%. Fullerenes C60: diameter ∼0.7 nm and 95.9% purity. The filler concentration comprised 5% wt. in all samples, with binary mixtures containing equal concentrations (2.5% wt.) of each constituent.

**Figure 1:**
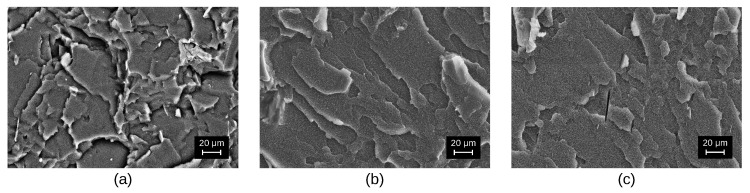
Cryo-SEM images of PS-based composites with binary fillers: C60 + Graphene (**a**), C60 + CNT (**b**) and CNT + Graphene (**c**).

The samples were fabricated by melt technology. Compositions based on polystyrene (PS) were prepared using a twin-screw micro compounder DSM Xplore 5 mL (Xplore Instruments BV, Sittard, Netherlands). Compounding was carried out at 275 ${}^{{}^{\circ}}C$ for 3–5 min at 140 rpm/min. To reduce oxidation, dry nitrogen was blown into the material supply zone at a rate of 500 mL/min. Block samples were fabricated by injecting the solution into a die heated to 50 ${}^{{}^{\circ}}C$. When removed from the microinjector, the die self-cooled down to room temperature in air. Block samples of composites of two types have been fabricated: plates 50×10×1.5 mm and blades with the working area of 20×4×1.5 mm. Samples made of pure PS have been fabricated for control purposes. Note that no significant agglomeration of filler particles has been observed at the concentration of 5% wt. See Figure 1 for cryo-SEM images of composites with binary nanofillers.

### 2.2. Analysis of Linear Elastic Properties

The linear elastic properties of the fabricated composite samples were studied by uniaxial tension, three-point bending test and dynamic mechanical analysis (DMA) by the three-point bending test.

The tensile tests were performed using an Instron 1122 (Norwood, MA, USA) universal testing instrument at the tensile speed of 10 mm/min. The tensile diagrams provided data on tensile strength $\sigma_{b}$, tensile strength at break $\epsilon_{b}$ and tensile modulus $E_{b}$. The Instron 1122 universal testing instrument was also used to perform the three-point bending tests. The load diagrams allowed for obtaining data on flexural stress $\sigma_{f}$, relative flexural strain $\epsilon_{f}$ and flexural modulus $E_{f}$.

DMA was performed using the dynamic mechanical analyzer DMA 242 C (Netzsch, Germany) operating in the frequency range of 0.1–50 Hz at the dynamic force of 2 N and temperature of 23 ± 2 ${}^{{}^{\circ}}C$. The frequency dependencies of the three parameters, elastic modulus $E^{\prime }$, loss modulus $E^{\prime \prime }$ and mechanical loss tangent tanδ have been determined.

### 2.3. Measurements of Nonlinear Elastic Moduli

**Figure 2:**
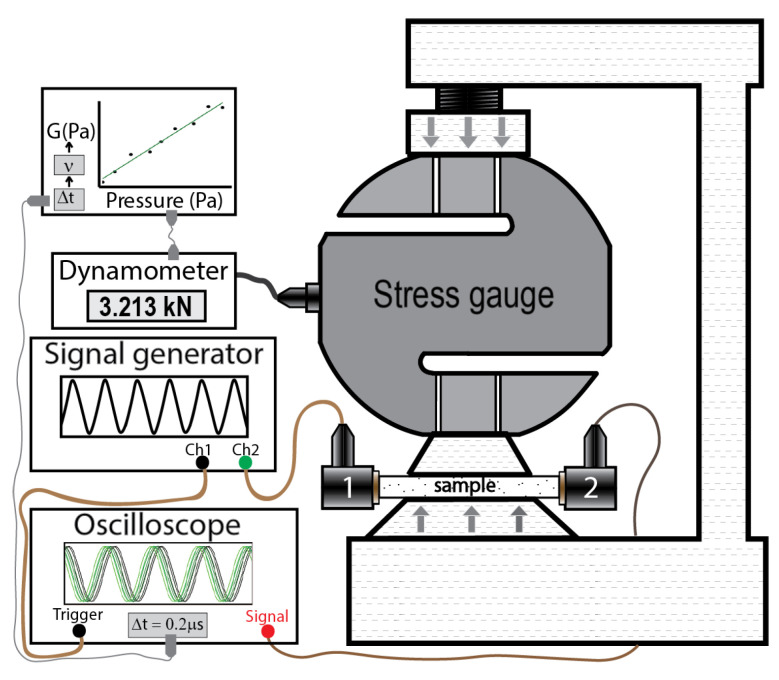
Experimental setup for measurements of ultrasound velocity as a function of applied stress.

The nonlinear elastic properties of the fabricated samples were analyzed by the values of nonlinear elastic (Murnaghan) moduli. For measurements, we used the approach first suggested in [23] and modified in our previous work [22]. The methodology is based on the acousto-elastic effect and utilizes measurements of longitudinal and shear ultrasonic wave velocities in the sample as a function of the applied static stress. The experimental setup is depicted schematically in Figure 2. The sample was clamped in the jaw vise; the applied pressure was manually increased gradually and controlled by a high-precision stress gauge. The applied pressure was varied between 0 and ≈8 MPa to minimize irreversible deformation of the samples and significant changes in their mechanical properties during the measuring process. An RTB2002 oscilloscope (Rohde&Schwarz, Munich, Germany) was used to record and analyze sinusoidal signals characterizing ultrasonic waves passing through the sample. Longitudinal waves were generated by piezoelectric transducers P121 (Amati Acoustics, St. Petersburg, Russia), and shear waves were generated by piezoelectric transducers V154-RB (Olympus, Center Valley, PA, USA). An AM300 Dual Arbitrary Generator (Rohde&Schwarz, Munich, Germany) provided the input signals with 7 V peak-to-peak amplitude and frequency of 1 MHz. The relative change in time required for ultrasonic waves to pass through the sample was determined by analyzing the recorded signals and their convolution with the signal recorded at zero pressure.

The dependencies of velocities of the three types of ultrasonic waves: longitudinal *p*-waves and transverse *s*-waves with displacement vectors orthogonal to each other, on the applied pressure provided data for determining the set of three Murnaghan moduli l,m and *n* [22,23]. The analysis was performed by considering the set of three Equations (1)–(3), taking into account some expansion of the sample occurring at increasing pressure (see [22] for details).

(1) $$\begin{array}{cccc} M_{x} & =\lambda +2\mu +\alpha_{x}T, & \;\alpha_{x} & =-\frac{2l-\frac{\lambda }{\mu }\left(2m+\lambda +2\mu \right)}{3\lambda +2\mu }, \end{array}$$

(2) $$\begin{array}{cccc} G_{y} & =\mu +\alpha_{y}T, & \;\alpha_{y} & =-\frac{m+\frac{\lambda }{4\mu }n+2\lambda +2\mu }{3\lambda +2\mu }, \end{array}$$

(3) $$\begin{array}{cccc} G_{z} & =\mu +\alpha_{z}T, & \;\alpha_{z} & =-\frac{m-\frac{\lambda +\mu }{2\mu }n-\lambda }{3\lambda +2\mu }, \end{array}$$

The equations clearly show linear dependencies of the effective elastic moduli $M_{x}$, $G_{y}$ and $G_{z}$ on the applied static pressure (*T*). Further evaluation of the corresponding slope coefficients $\alpha_{x}$, $\alpha_{y}$ and $\alpha_{z}$ by least square fitting of the experimental data allows for determining the three nonlinear elastic moduli *l*, *m* and *n* using the set of equations, with the Lame moduli λ, μ obtained from measurements of longitudinal and shear wave velocities at zero pressure:

(4) $$\begin{array}{c} \;\;\;l=-\frac{3\lambda +2\mu }{2}\alpha_{x}-\frac{\lambda (\lambda +\mu )}{\mu }\left(1+2\alpha_{y}\right)+\frac{\lambda^{2}}{2\mu }\left(1-2\alpha_{z}\right), \end{array}$$

(5) $$\begin{array}{c} \;\;\;m=-2\left(\lambda +\mu \right)\left(1+\alpha_{y}\right)+\lambda \left(1-\alpha_{z}\right), \end{array}$$

(6) $$\begin{array}{c} \;\;\;n=-4\mu \left(1+\alpha_{y}-\alpha_{z}\right). \end{array}$$

**Figure 3:**
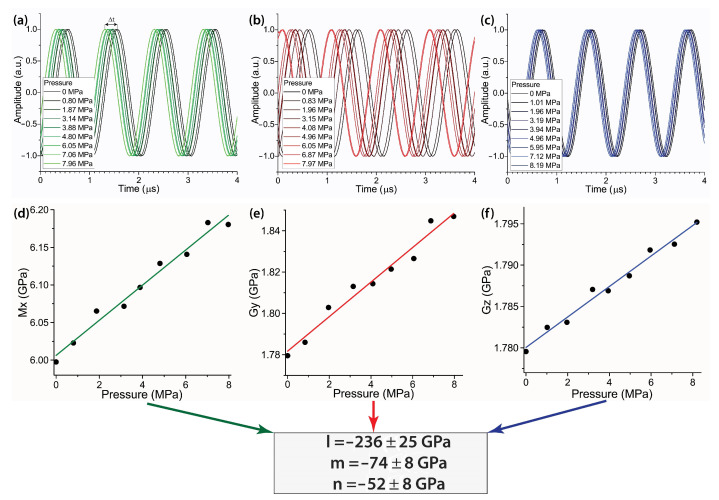
Illustration of the procedure aimed to determine the nonlinear elastic moduli of a composite sample on the example related to the polystyrene sample filled with 2.5% wt. graphene and 2.5% wt. C${}_{60}$ nanoparticles. (**a**–**c**) detection of ultrasonic waves and evaluation of time delay induced by the application of static pressure onto the sample: (**a**) longitudinal waves, (**b**) shear waves perpendicular to the applied pressure and (**c**) shear waves parallel to the applied pressure. (**d**–**f**) calculated dependencies of effective elastic moduli: (**d**) M${}_{x}$, (**e**) G${}_{y}$ and (**f**) G${}_{z}$ on the applied pressure. The calculated Murnaghan moduli are shown in the bottom row.

The workflow on the determination of nonlinear elastic moduli is illustrated schematically in Figure 3 for the example of the PS sample with binary inclusions of graphene and C${}_{60}$ nanoparticles. A series of sinusoidal signals characterizing longitudinal and shear waves at various static pressures applied to the sample demonstrate variations in the time Δt required for the ultrasonic wave to propagate through the sample at the increasing static pressure (Figure 3a–c). Taking into account values of longitudinal and shear wave velocities obtained at normal conditions (zero pressure), a linear dependence between the nonlinear effective moduli $M_{x}$, $G_{y}$ and $G_{z}$ can be extracted, and slope coefficients $\alpha_{x}$, $\alpha_{y}$ and $\alpha_{z}$ can be evaluated (Figure 3d–f). The determined coefficients were further used for the calculation of nonlinear elastic moduli using Equations (4)–(6) (see the bottom row in Figure 3).

Since the samples were fabricated in the form of plates 1.5 mm thick, due to the difficulty of conducting ultrasonic experiments with so thin samples, three plates of the same material were bonded together to make a sandwich with a larger cross-section. This allowed us to increase the working area on the interface between the sample and piezoelectric transducers and to provide a reliable signal. For bonding, we used the ethylcyanoacrylate adhesive Superglue, which, as we have previously shown [22,24,25], does not cause changes in the nonlinear elastic properties of polystyrene samples. The resulting sandwich samples had a size of 5×10×50 mm.

## 3. Results and Discussion

### 3.1. Linear Elastic Properties of PS-Based Nanocomposites

**Figure 4:**
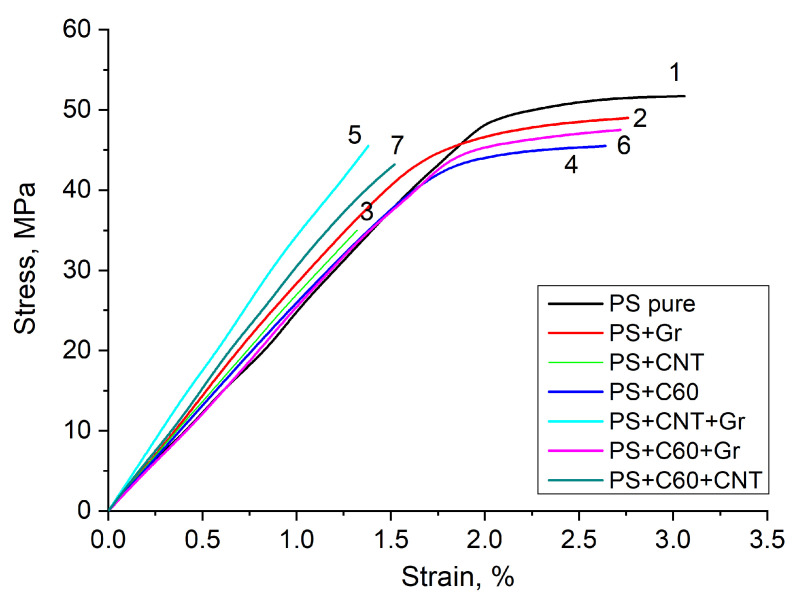
Tensile stress-strain curves of PS-based nanocomposites with the addition of single-type particles (graphene, C${}_{60}$ and CNT) and binary mixtures (graphene + C${}_{60}$, C${}_{60}$ + CNT, CNT + graphene): 1—PS pure, 2—PS + Gr, 3—PS + CNT, 4—PS + C${}_{60}$, 5—PS + CNT + Gr, 6—PS + C${}_{60}$ + Gr, 7—PS + C${}_{60}$ + CNT.

Figure 4 presents tensile stress-strain diagrams of the PS-based nanocomposites and pure PS. The data obtained from these diagrams on the tensile strength $\sigma_{b}$, tensile strain at break $\epsilon_{b}$ and tensile modulus $E_{b}$ are summarized in Table 1. The data on the flexural stress $\sigma_{f}$, relative flexural strain $\epsilon_{f}$ and flexural modulus $E_{f}$ obtained from load diagrams of the three-point bending tests are also shown in Table 1.

As can be seen from Figure 4 and Table 1, the samples demonstrated significantly different tensile behavior. Composites containing carbon nanotubes both as a single-type filler and as a constituent of the binary mixture demonstrated brittle fracture. The strain at break of these samples was about 2 times lower than that of other samples, which indicates a better adhesion of CNTs to the polystyrene matrix [26]. For other samples, this parameter is 20–25% lower than that for pure PS. This reduction in the strain at break is close to Nielsen’s model, which predicts a reduction by $\phi^{1/3}\approx 27\%$, where ϕ≈2% is the volume fraction of inclusions [26]. In all the composite samples, the tensile modulus $E_{b}$ was higher than that in pure PS, with the highest rise (of 50%) obtained in the sample with the binary mixture of CNT and graphene.

Since the samples of PS-based composites were sufficiently flexible, and no brittle fracture was observed during bending tests, the flexural stress limit was calculated at a given deflection value of S=6 mm. However, it is worth noting that the samples containing carbon nanotubes as a single filler or as a constituent in a binary mixture showed more fragile behavior at stronger bending. Flexural tests have shown that flexural stress $\sigma_{f}$ rose most noticeably for the sample with a binary mixture of CNT and graphene. The same tendency was observed for the flexural modulus $E_{f}$, which exhibited a 50% rise in this sample (in 2 GPa). PS filling with graphene solely and the binary mixture of C${}_{60}$ and CNT provided a similar rise in the flexural stress and flexural modulus by about 9% and 20%, respectively.

**Figure 5:**
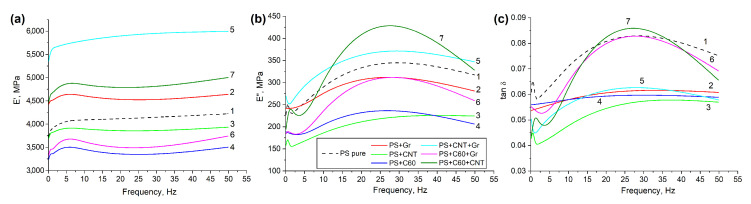
Frequency dependencies of the elastic modulus $E^{\prime }$ (**a**), loss modulus $E^{\prime \prime }$ (**b**) and mechanical loss tangent tanδ (**c**) of pure polystyrene and composites with single-type nanoparticles and binary mixtures: 1—PS pure, 2—PS + Gr, 3—PS + CNT, 4—PS + C${}_{60}$, 5—PS + CNT + Gr, 6—PS + C${}_{60}$ + Gr, 7—PS + C${}_{60}$ + CNT.

Figure 5 presents the frequency dependencies of the elastic modulus $E^{\prime }$, loss modulus $E^{\prime \prime }$ and mechanical loss tangent tanδ of the samples obtained by DMA analysis. As can be seen in Figure 5, the elastic modulus practically did not depend on frequency in all samples. The highest value of the elastic modulus was obtained in the sample with a binary filling of CNT and graphene, the rise over that for pure PS comprised about 35%. Surprisingly, the elastic moduli of PS + C${}_{60}$, PS + CNT and PS + C${}_{60}$ + Gr were below those of pure PS. The loss modulus had a clear maximum at the frequency of 25–30 Hz in almost all the samples, which can be associated with an increase in the segmental mobility in the polymer matrix. The loss modulus was higher than that of pure PS only in composites with binary mixtures containing CNTs. The loss tangent for all the composites was significantly lower than that for pure PS within the entire frequency range except for the sample of PS + C${}_{60}$ + CNT in the range of ≈15–40 Hz. The graphs clearly demonstrate an increase in both the elastic and viscous components of the modulus in the samples containing binary mixtures of extended and layered particles (CNT + Gr) and extended and spherical particles (CNT + C60). The introduction of the mixture of layered and spherical particles (Gr + C60) and single-type fillers led to the decrease in both moduli as compared to those of pure PS, except for the elastic modulus with CNTs, which showed a slight increase over that of pure PS. Most likely, this behavior is due to the formation of the percolation network in the bulk of the polymer matrix in the presence of extended particles. It was suggested that in composites with hybrid CNT + Gr fillers, carbon nanotubes can embed graphene particles and bridge them into an interconnected network (see [5] and references therein). This network may provide more even distribution of the load applied to the composite sample. In the absence of such a network, independent particles may rather act as defects, loosening the structure of the polymer matrix. Note that the observed effects require further, more detailed investigations.

**Figure 6:**
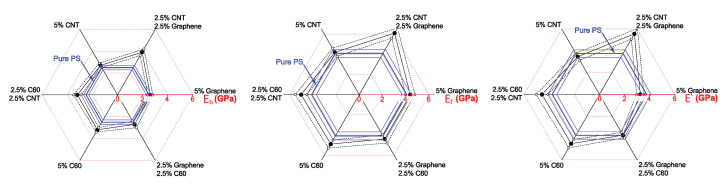
Graphical representation of the data on measured linear elastic moduli (E${}_{b}$, E${}_{f}$, E′) of the pure polystyrene sample (shown in blue) and polystyrene with the addition of single-type inclusions (graphene, C${}_{60}$, CNT) and binary mixtures (C${}_{60}$ + Gr, C${}_{60}$ + CNT, CNT + Gr) of nanoparticles. Both measured values (filled circles and bold lines) and 68% confidence intervals (dashed lines and empty circles) are indicated.

Figure 6 presents a graphical representation of the data on linear elastic moduli $E_{b}$, $E_{f}$ and $E^{\prime }$ of pure PS and composite samples obtained from tensile, flexural and DMA tests. As can be clearly seen, all three moduli demonstrated similar dependencies on filler composition. The maximal values were observed in composites filled with the binary mixture of CNT and graphene. A smaller increase was observed in composites filled with C${}_{60}$ and C${}_{60}$ + CNT. Other fillers did not cause noticeable changes in the linear elastic moduli.

The changes in linear elastic properties of PS-based composites with carbon allotropes observed in this work are in good correspondence with the data obtained by other authors, see, e.g., [27,28,29]. In particular, the introduction of single-type nanoparticles into a polystyrene matrix led to an increase in the elastic modulus from 20% to 70%, depending on the filler concentration. At the same time, the formation of an intercalated structure of PS-Graphene composites was shown to improve the mechanical characteristics of the material at lower filler concentrations due to a better transfer of load between the matrix and filler and the formation of a less defective structure [29]. A number of research groups have also shown that the elastic moduli of PS-CNT composites increased only slightly over those of pure PS, see, e.g., [30]. Several groups observed a synergistic effect of graphene and carbon nanotubes on the mechanical properties of polymer composites [31,32]. It was suggested that a synergistic effect can be achieved with hybrid nanofillers of different geometrical shapes. The combination of such fillers can form interconnected structures, improving the dispersion of filler particles and changing the crystallinity of the polymer matrix.

### 3.2. Nonlinear Elastic Moduli

The obtained data on nonlinear elastic moduli for each composite sample are summarized in Table 2. In spite of relatively high measurement errors of moduli values (median error ≈17%, average error ≈25%), an enhancement of nonlinear elastic properties can be clearly observed both with the addition of any single type of nanoparticles or their binary mixtures. In general, the most significant change in nonlinear elastic moduli was observed in PS samples filled with 5% wt. of fullerene C${}_{60}$ nanoparticles. Significant changes were also obtained in the sample filled with the binary mixture of CNT and graphene. This hybrid filler also provided considerable changes in linear elastic moduli E${}_{b}$ and E${}_{f}$ (see Table 1), while fullerenes solely caused only a slight increase in both tensile and flexural moduli. Note that both linear and nonlinear elastic parameters of samples with binary fillings can not be estimated just by averaging the values obtained for the samples filled with each type of nanoparticle solely. Therefore, a specific synergistic effect can be observed when the polymer sample is filled with a mixture of different nanoparticles, which can be a result of their direct or indirect interaction inside the polymer matrix.

**Figure 7:**
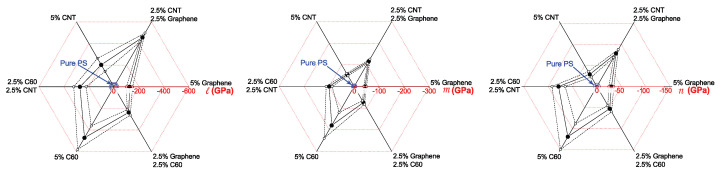
Representation of the data on measured nonlinear elastic moduli (*l*,*m*,*n*) of the pure polystyrene sample (shown in blue) and polystyrene with the addition of single-type inclusions (graphene, C${}_{60}$, CNT) and binary mixtures (C${}_{60}$ + Gr, C${}_{60}$ + CNT, CNT + Gr) of nanoparticles. Both measured values (filled circles and bold lines) and 68% confidence intervals (dashed lines and empty circles) are indicated. The graphs clearly show a significant increase in nonlinear elastic properties of all the composites over pure PS. It is also clearly seen that the nonlinear elastic moduli of samples with binary fillers are not equal to the average values of those of samples with single-type nanofillers.

Figure 7 presents a graphical representation of the data on nonlinear elastic moduli *l*, *m* and *n* of pure PS and composite samples obtained from ultrasonic measurements. As can be seen from Figure 6 and Figure 7, all types of inclusions provided considerably more pronounced changes in the nonlinear elastic characteristics than in the linear ones. In particular, the highest rise of nonlinear moduli *l*, *m* and *n* in 16, 22 and 34 times, respectively, was observed in the sample filled with fullerenes. While the maximal rise of linear moduli observed in the sample with CNT + Gr was only of the order of 50%. The observed effect of nanoinclusions on nonlinear moduli is much larger than those that can be predicted by the micromechanical model [33], which will be studied in more detail in further investigations. Note that the experiments reported in this paper were carried out on samples fabricated at a different temperature regime than in our previous work [22], and measurements of nonlinear elastic moduli were performed at another ultrasound frequency. As we have previously shown, the temperature regime used for sample fabrication and ultrasound frequency used for measurements of nonlinear elastic moduli can significantly affect the moduli values [25,34]. Thus the somewhat difference in values obtained in this work and in ref. [22] can be due to different experimental conditions.

## 4. Conclusions

In this work, we reported on the fabrication and a comprehensive investigation of both linear and nonlinear elastic properties of polystyrene-based nanocomposites filled with the same weight amounts of single-type and binary mixtures of allotropic forms of carbon nanoparticles. It was shown that the most significant variations in linear and nonlinear elastic properties occurred in different nanocomposites. In particular, the most pronounced enhancement in linear elastic moduli (in about 50%) obtained in tensile, flexural and DMA tests was recorded in the sample filled with spherical fullerene nanoparticles. While the most profound rise in absolute values of nonlinear elastic moduli (in tens of times) was obtained in the sample filled with the mixture of carbon nanotubes and graphene. The analysis of both linear and nonlinear elastic properties of composites has shown that, in general, the elastic characteristics of samples with binary mixtures containing equal concentrations of each nanofiller can not be estimated by the averaging of values obtained in composites with these types of fillers solely. The observed tendency demonstrates the synergistic effect of fillers of different dimensionality on the elastic properties of nanocomposites.

## Figures and Tables

**Table 1:** Linear elastic parameters of PS-based nanocomposites obtained from tensile and flexural tests.

Sample	Tensile			Flexural		
	$\sigma_{b}$, MPa	$\epsilon_{b}$, %	$E_{b}$, GPa	$\sigma_{f}$, MPa	$\epsilon_{f}$, %	$E_{f}$, GPa
PS pure	52±5	3.3±0.5	2.5±0.2	80±7	2.0±0.2	4.1±0.4
PS + C${}_{60}$	46±3	2.5±0.3	2.6±0.2	78±4	2.0±0.2	4.4±0.4
PS + CNT	36±3	1.4±0.2	2.7±0.3	82±5	2.0±0.2	4.4±0.4
PS + Gr	48±3	2.7±0.3	3.2±0.2	87±4	2.0±0.2	4.9±0.4
PS + C${}_{60}$ + CNT	43±4	1.5±0.4	3.2±0.3	87±5	2.0±0.2	5.0±0.5
PS + C${}_{60}$ + Gr	48±4	2.7±0.4	2.7±0.2	80±4	2.0±0.2	4.2±0.4
PS + CNT + Gr	46±4	1.4±0.3	3.9±0.3	95±4	2.0±0.2	6.1±0.5

**Table 2:** Nonlinear elastic moduli of PS-based nanocomposites obtained from ultrasonic measurements.

Sample	ρ (g/cm${}^{3}$)	*l* (GPa)	*m* (GPa)	*n* (GPa)
PS pure	1.00	−29 ± 10	−8 ± 4	−3.5 ± 1.4
PS + C${}_{60}$	1.04	−471 ± 178	−179 ± 59	−119 ± 30
PS + CNT	1.07	−203 ± 88	−55 ± 5	−29 ± 6
PS + Gr	1.11	−124 ± 24	−44 ± 5	−30 ± 5
PS + C${}_{60}$ + CNT	1.05	−271 ± 49	−99 ± 11	−78 ± 14
PS + C${}_{60}$ + Gr	1.06	−236 ± 24	−74±8	−53 ± 7
PS + CNT + Gr	1.09	−456 ± 40	−116 ± 10	−79 ± 9

## Data Availability

The data are available within the paper.
